# Ischemic and hemorrhagic abdominal complications in COVID-19 patients: experience from the first Italian wave

**DOI:** 10.1186/s40001-022-00793-x

**Published:** 2022-08-31

**Authors:** Pietro Andrea Bonaffini, Paolo Niccolò Franco, Alice Bonanomi, Cinzia Giaccherini, Clarissa Valle, Paolo Marra, Lorenzo Norsa, Marina Marchetti, Anna Falanga, Sandro Sironi

**Affiliations:** 1 Department of Radiology, ASST Papa Giovanni XXIII Hospital, Piazza OMS 1, 24127 Bergamo, BG Italy; 2 School of Medicine, University of Milan-Bicocca, Piazza dell’Ateneo Nuovo 1, 20126 Milan, MI Italy; 3 Unit of Immuno-Hematology and Transfusion Medicine and Center of Hemostasis and Thrombosis, Papa Giovanni XXIII Hospital, Piazza OMS 1, 24127 Bergamo, BG Italy; 4 Unit of Pediatric Hepatology Gastroenterology and Transplantation, Papa Giovanni XXIII Hospital, Piazza OMS 1, 24127 Bergamo, BG Italy

## Abstract

**Purpose:**

To report ischemic and haemorrhagic abdominal complications in a series of COVID-19 patients. To correlate these complications with lung involvement, laboratory tests, comorbidities, and anticoagulant treatment.

**Methods:**

We retrospectively included 30 COVID-19 patients who undergone abdomen CECT for abdominal pain, between March 16 and May 19, 2020. Ischemic and haemorrhagic complications were compared with lung involvement (early, progressive, peak or absorption stage), blood coagulation values, anticoagulant therapy, comorbidities, and presence of pulmonary embolism (PE).

**Results:**

Ischemic complications were documented in 10 patients (7 receiving anticoagulant therapy, 70%): 6/10 small bowel ischemia (1 concomitant obstruction, 1 perforation) and 4/10 ischemic colitis. Main mesenteric vessels were patent except for 1 superior mesenteric vein thrombosis. Two ischemia cases also presented splenic infarctions. Bleeding complications were found in 20 patients (all receiving anticoagulant treatments), half with active bleeding: hematomas in soft tissues (15) and retroperitoneum (2) and gastro-intestinal bleeding (3). Platelet and lymphocyte were within the normal range. d-Dimer was significantly higher in ischemic cases (*p* < 0.001). Most of the patients had severe lung disease (45% peak, 29% absorption), two patients PE.

**Conclusions:**

Ischemic and haemorrhagic abdominal complications may occur in COVID-19 patients, particularly associated to extended lung disease. CT plays a key role in the diagnosis of these potentially life- threatening conditions.

## Introduction

COVID-19 mainly affects the respiratory system, with involvement ranging from flu-like symptoms (weakness, cough, fever) to interstitial pneumonia, acute respiratory distress syndrome (ARDS) and respiratory failure. However, it is now widely accepted that all the other systems can be potentially affected, including the gastrointestinal (GI) tract and abdominal solid organs [1, 2].

The pathogenesis of the multi-organ involvement is still under investigation, but likely includes direct viral toxicity, endothelial cell damage and thromboinflammation, immune response and renin–angiotensin–aldosterone system (RAAS) dysregulation [3, 4]. In particular, the angiotensin-converting enzyme 2 (ACE2) has been identified to be the major entry receptor for SARS-CoV-2 in human cells [5]. ACE2 is predominantly expressed by lung vascular endothelial cells, but also in extrapulmonary tissues, including heart, nervous system, and GI tract [1, 6, 7]. By invading cells, SARS-CoV-2 causes both a direct damage and RAAS dysregulation. This last alteration contributes to the endothelial damage, that is one of the major responsible for the formation of microthrombi and subsequent ischemic manifestations of COVID-19 [8, 9]. The intense inflammatory state elicited by SARS-CoV-2 infection also leads to a severe hemostatic system derangement, with systemic activation of blood coagulation and high risk of thrombosis. This viral infection induces an excessive and aberrant hyper-inflammatory host immune response (“cytokine storm”) [10]. High levels of pro-inflammatory cytokines cause extensive tissue damage and an intense activation of the procoagulant properties of the vascular endothelium as well as of platelets and leukocytes.

The most commonly reported COVID-19 GI symptoms are nausea, diarrhea, ischemic and bleeding complications [6, 11–14], even in patients without pulmonary involvement [13]. Bleeding complications had been detected not only in the GI tract, but also in other sites, especially in soft tissues [15]. These complications can occur in COVID-19 patients as part of the virus-induced coagulopathy or as a complication of anticoagulant treatments for management or prevention of either pulmonary embolism or more common thrombotic coagulopathy [1]. Indeed, the International Society on Thrombosis and Hemostasis (ISTH) recommends offering prophylactic anticoagulation with low molecular weight heparins (LMWH) to all hospitalized COVID-19 patients as early as possible to prevent thrombotic events and organ damage [16].

On these bases, the aim of this study was to describe the occurrence of ischemic and/or hemorrhagic abdominal complications detected on CT scans, in COVID-19 patients undergoing imaging investigation for abdominal pain. Moreover, we investigated any possible correlations of these clinical scenarios with lung involvement, patient comorbidities, blood tests, and anticoagulant therapy.

## Methods

The ethical committee approval for this retrospective monocentric observational study (protocol ABDOCOVID) was obtained from the local committee of Papa Giovanni XXIII Hospital, Bergamo. Need for informed consent was waived due to the pandemic contingency.

### Study population

We included consecutive patients with suspected COVID-19-related symptoms and then confirmed SARS-CoV-2 positive, either hospitalized or recently admitted in the emergency department (ED) at a single tertiary Hospital, between March 16 and May 19, 2020. All the abdominal CT performed in the reference period were reviewed. The inclusion criteria were as follows: (a) positive real-time polymerase chain reaction (RT-PCR) in throat swab samples; (b) acute abdominal pain and/or clinical suspicion of intestinal ischemia or bleeding; (c) contrast-enhanced abdominal CT. The exclusion criteria were: (a) no adequate diagnostic imaging data (i.e., CT scans performed without contrast or in outside institutions); (b) positive RT-PCR obtained more than 3 weeks before the CT scan.

### Clinical and laboratory data

Prothrombin time (PT)–international normalized ratio (INR), activated partial thromboplastin time (aPTT), fibrinogen, platelets, neutrophiles and lymphocytes count, C-reactive protein (CRP), interleukin-6 (IL-6), procalcitonin and d-dimer (DD) levels were collected. We considered parameters obtained within a maximum interval of 2 days compared to CT acquisition. Data available about oral or parenteral anticoagulant therapies and patients’ comorbidities (cardiovascular, metabolic, respiratory, others) were also annotated.

### CT acquisition and image analysis

*CT study protocol* CT abdominal scans were acquired with patients supine, on a 64-slice scanner (Brilliance 64-slice—Philips Medical Systems, Best, Netherlands; Revolution EVO—GE Medical Systems, Chicago, IL, Unites States). The unenhanced acquisition was followed by scanning during and after the intravenous bolus injection of a non-ionic contrast medium (1.2 mL/kg; flow rate at least 3 mL/s; iomeprol 350 mg/mL or 400 mg/mL; Bracco Imaging); a rapid saline solution flush followed. Chest CT scans performed simultaneously or close to the abdominal acquisition were analyzed. All the acquisitions were performed with the following parameters: tube voltage, 100–120 kVp; automatic exposure control for tube current; pitch 0.6–1.2; collimation 12–40 mm. Images were reconstructed with 1.5–2 mm slice thickness. Chest CT images were reconstructed with 0.9–1.5 mm slice thickness using standard lung window settings (width, 1600 HU and level, − 600 HU).

*Images analysis* The abdominal CT scans were evaluated in consensus by two reviewers (PAB and PM, two radiologists with 11 and 7 years of experience, respectively). Radiological signs of abdominal ischemia (bowel wall hypoenhancement/thickening, mesenteric edema, parietal pneumatosis, porto-mesenteric venous gas, pneumoperitoneum, parenchymatous organs infarction) or bleeding complications (hyperdense fluid collections, active contrast blush) were reported.

Chest images analysis was independently performed by two radiology residents (FPN and AB, with 3 and 2 years of chest imaging experience, respectively). For the final diagnosis of parenchymal stage, the evaluation of the most experienced reader was considered. For lung disease involvement a stage was assigned, according to Pan et al. [17]: early (ground-glass opacities/GGO and crazy paving pattern, maximum three lobes involved), progressive (> 3 involved lobes, initial consolidations), peak (prevalent consolidations) and absorption (gradual resolution).

### Statistical analysis

Categorical data were summarized as frequencies and proportion, while continuous variables as median and 25–75th percentile range. Differences in distribution of categorical variables were tested by *χ*^2^ test. Differences between groups of continuous variables were tested by Student *t* test (if normal distribution) or by the non-parametric Mann–Whitney test. Statistical analysis was performed with SPSS v21.0 (IBM Corp).

## Results

### Patients, outcome, chest, and abdominal imaging findings

In the period between March 16^th^ and May 19^th^, 2020, a total of 250 patients (74 females; mean age 52.4 years, range 1–88) underwent abdominal CT, in emergency care setting (*n* = 67) or during hospitalization (*n* = 183). In this total cohort, 113 patients (42.2%) had a positive RT-PCR for SARS-CoV-2. Among these, only 30 patients (12%) fulfilled the inclusion and exclusion criteria of the study: positive COVID-19 test with abdominal pain and who underwent CECT of the abdomen for this clinical reason. The majority of patients were male (63%) and the median age was 68.5 years (range 48–88): 22/30 patients underwent CT during hospitalization in medical wards (median hospitalization time 19 days; 4–163) and 8/30 during ED permanence, before discharge or ward admittance (Table 1). The results of abdominal CT scans revealed radiological signs of ischemia in 10 patients (33%) and bleeding in 20 cases (67%): 6/10 ischemic complications occurred in emergency care setting, while 18/20 bleeding cases occurred in hospitalized patients.

**Table 1:** Main CT findings of included COVID positive patients (30)

Patient#	Sex	Age	Hospitalization /ED	Ischemia /bleeding	CT findings	Additional sites of abdominal ischemia	Main abdominal vein thrombosis	Active bleeding	Embolization
001	M	85	ED	Ischemia	Ischemic colitis	None	None	None	None
002	M	55	Hospitalized	Ischemia	Small bowel ischemia with intestinal obstruction	None	None	None	None
003	M	69	ED	Ischemia	Ischemic colitis	None	None	None	None
004	M	56	Hospitalized	Ischemia	Small bowel ischemia	Spleen	None	None	None
005	F	79	ED	Ischemia	Small bowel ischemia	None	None	None	None
006	M	63	Hospitalized	Ischemia	Ischemic colitis	Spleen	None	None	None
007	M	57	ED	Ischemia	Small bowel ischemia	None	None	None	None
008	F	83	Hospitalized	Ischemia	Small bowel ischemia with intestinal perforation	None	None	None	None
009	M	52	ED	Ischemia	Ischemic colitis	None	None	None	None
010	M	62	Hospitalized	Ischemia	Small bowel ischemia	Kidneys	SMV	None	None
011	M	66	ED	Bleeding	GI bleeding	None	None	None	None
012	F	59	Hospitalized	Bleeding	Hematoma in iliopsoas	None	None	None	None
013	M	63	Hospitalized	Bleeding	GI bleeding	None	None	None	None
014	M	62	Hospitalized	Bleeding	Retroperitoneal hematoma	None	None	Yes	Yes
015	M	48	Hospitalized	Bleeding	GI bleeding	None	None	Yes	None
016	M	50	Hospitalized	Bleeding	Hematoma in iliopsoas	None	None	Yes	None
017	F	68	Hospitalized	Bleeding	Hematoma in rectus abdominis	None	None	None	None
018	M	57	Hospitalized	Bleeding	Hematoma in iliopsoas	None	None	None	None
019	M	75	Hospitalized	Bleeding	Hematoma in iliopsoas	None	None	None	None
020	M	54	Hospitalized	Bleeding	Hematomas in multiple muscles	None	None	Yes	None
021	F	77	Hospitalized	Bleeding	Hematoma in iliopsoas	None	None	Yes	Yes
022	F	71	Hospitalized	Bleeding	Hematomas in multiple muscles	None	None	None	None
023	F	80	Hospitalized	Bleeding	Hematoma in iliopsoas	None	None	None	None
024	F	81	ED	Bleeding	Hematoma in rectus abdominis	None	None	Yes	Yes
025	M	69	Hospitalized	Bleeding	Hematomas in multiple muscles	None	None	None	None
026	F	88	Hospitalized	Bleeding	Hematoma in iliopsoas	None	None	Yes	None
027	F	69	ED	Bleeding	Retroperitoneal hematoma	None	None	Yes	Yes
028	M	79	Hospitalized	Bleeding	Hematoma in iliopsoas	None	None	None	None
029	F	77	Hospitalized	Bleeding	Hematoma in iliopsoas	None	None	Yes	Yes
030	M	81	Hospitalized	Bleeding	Hematoma in rectus abdominis	None	None	Yes	None

**Fig. 1:**
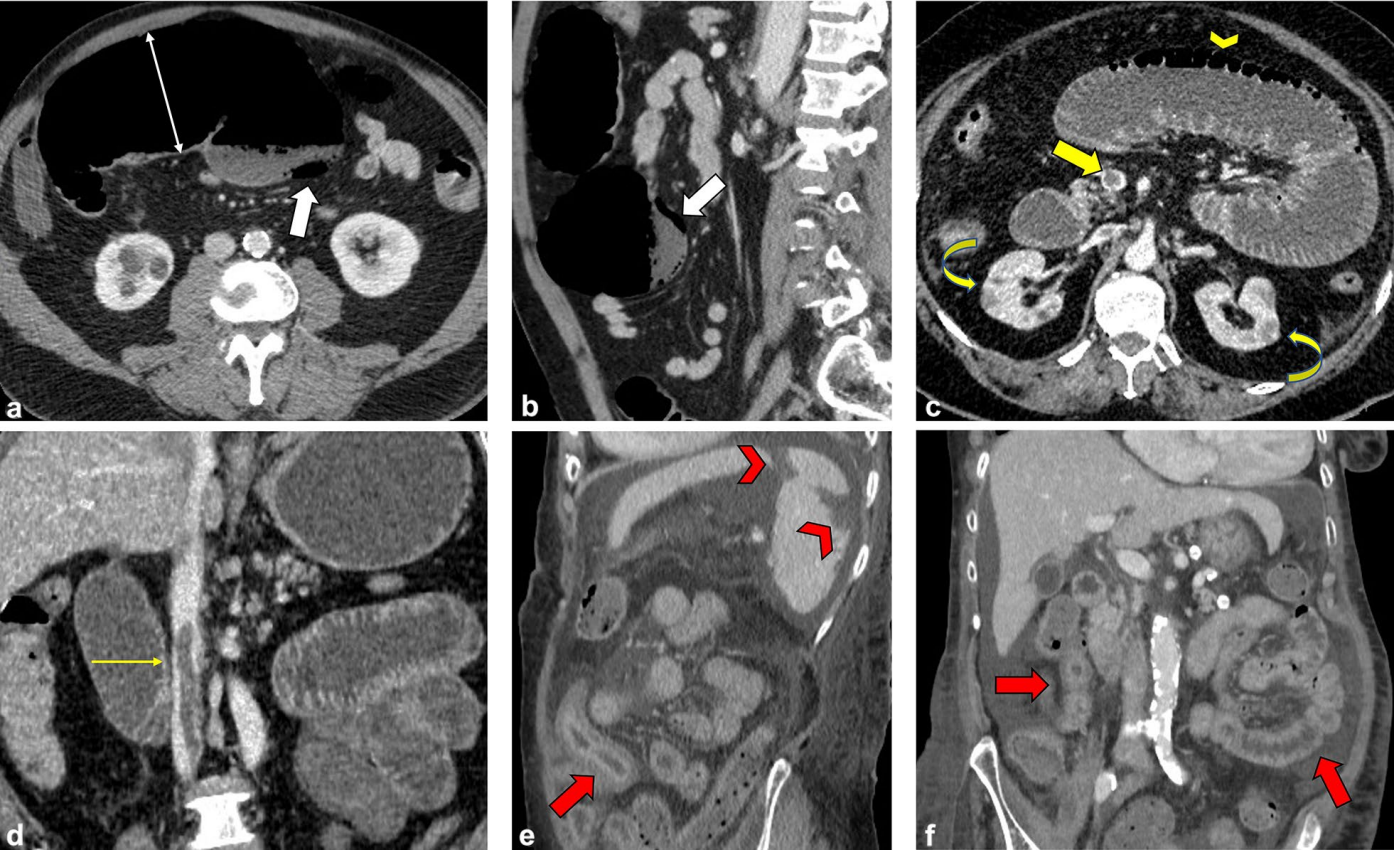
**Representative images of abdominal ischemic complications.** Computed tomography scans from COVID-19 patients with ischemic complications. **A**, **B** Axial and sagittal CT images of a 63-year-old patient show evidence of ischemic colitis involving the transverse colon which looks overdistended (white thin arrow, **A**), with thin non-enhancing walls and evidence of pneumatosis coli (white arrows, **A** and **B**). The main mesenteric vessels were patent (not shown). **C**, **D** Axial and coronal CT images of a 62-year-old patient revealed superior mesenteric vein thrombosis (yellow arrow, **C**) and dilated small bowel loops with pneumatosis intestinalis (yellow arrowhead, **C**). Concomitant signs of renal infarctions were also noted (yellow curved arrows, **C**). The same patient also showed inferior cava vein thrombosis (thin yellow arrow, **D**). **E**, **F** Sagittal and coronal CT images of a 79-year-old patient with markedly thickened and layered small bowel walls (red arrows, **E** and **F**) associated with multiple peripheral splenic infarcts (red arrowheads, **E**) with areas of mottled increased attenuation; the main splenic vessels were patent (not shown)

Specifically, the 10 ischemic complications included 4 cases of colitis (2 cases of ischemic colitis confirmed by endoscopy) and 6 of small bowel ischemia (Fig. 1). Among these last patients, we found 1 concomitant small bowel obstruction (SBO) and 1 perforation. The main mesenteric vessels were patent except in one case with superior mesenteric vein (SMV) thrombosis (Table 1). Two patients with ischemia presented concurrent splenic infarctions and one patient also showed bilateral renal infarction. ED: emergency department. GI: gastrointestinal.

**Fig. 2:**
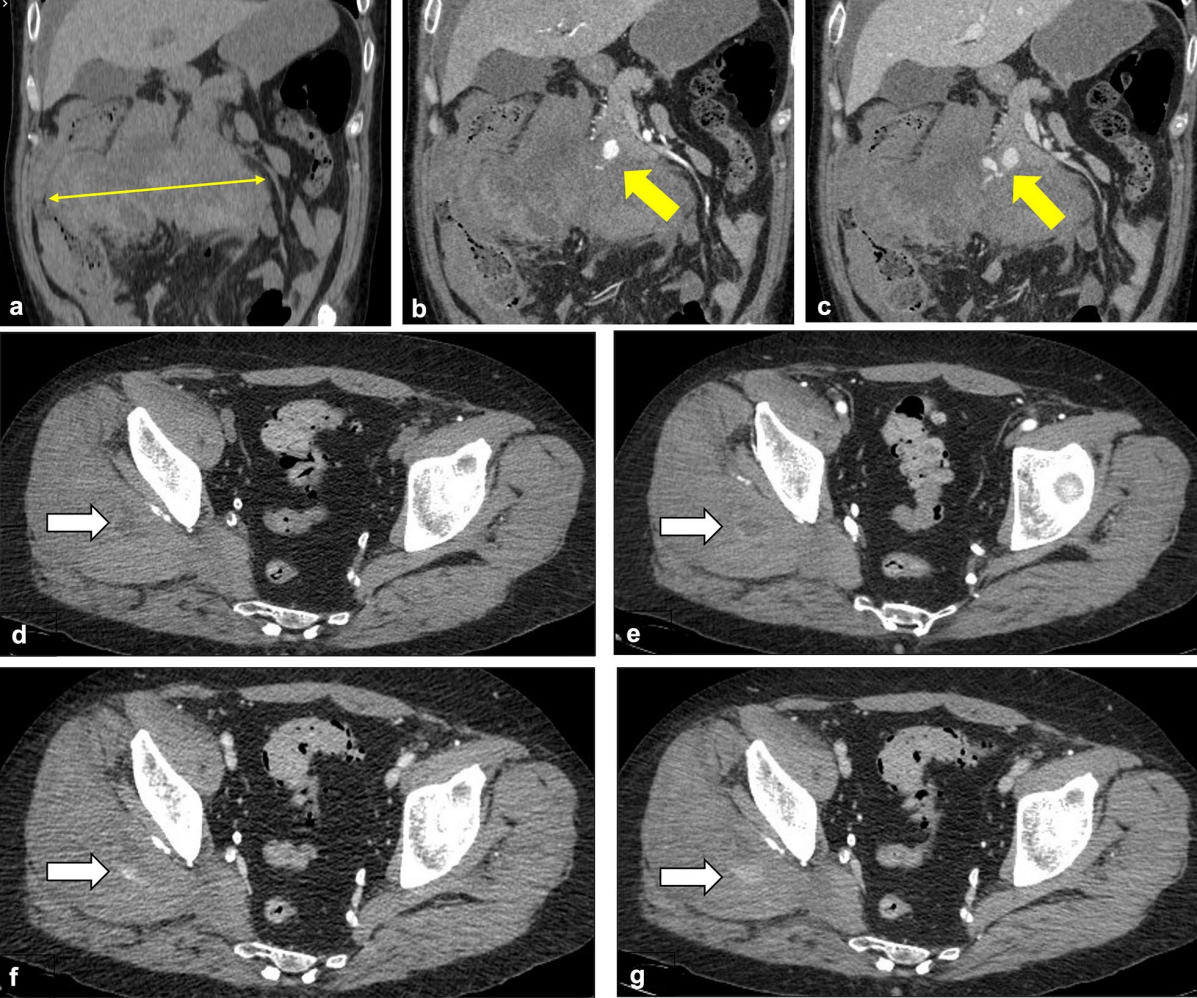
**Representative images of hemorrhagic complications in the abdomen.** CT scans from COVID-19 patients with hemorrhagic complications. **A**, **B**, **C** Coronal contrast-enhanced CT images of a 62-year-old patient show a large retroperitoneal hematoma (thin yellow arrow, **A**), with arterial contrast blush from pancreaticoduodenal artery (yellow arrows, **B** and **C**). **D**, **E**, **F**, **G** Axial contrast-enhanced CT scans of a 54-year-old patient revealed a ill-defined hematoma within the right gluteus medium muscle (white arrow in **D**), with active bleeding in arterial, venous and delayed phase (white arrows, **E**, **F** and **G**)

The 20 bleeding manifestations were mainly represented by spontaneous hematomas in soft tissues, which were present in 15/20 patients (75%): 9 in iliopsoas muscles, 3 in rectus abdominis muscles and 3 in multiple abdominal and/or thoracic muscles (Fig. 2). The remaining 5 cases included 3 upper GI bleeding (15%) and 2 retroperitoneal hematomas (10%). Radiological signs of active hemorrhage were observed in 10/20 cases: seven intra-muscular, two retroperitoneal (one from pancreaticoduodenal and one from lumbar arteries) and one intra-peritoneal bleeding. Five patients with active hemorrhage (3 with intramuscular and 2 with retroperitoneal hematomas), underwent angiographic procedures within few hours from the CT acquisition: one source of active bleeding was not identified during angiography.

Twenty-four patients also performed a chest CT: 13 simultaneously and 11 within maximum before or after 2 weeks compared to the abdominal scan. Parenchymal stages were assessed as follows: 11 peak (45.8%), 7 absorption (29.2%), 5 progressive (20.8%) and 1 early (4.2%). The interobserver agreement for assigning parenchymal stages was substantial (87.5% of agreement; Cohen’s k: 0.742).

A correlation between ischemic or hemorrhagic events with hospital staying was observed (*χ*^2^ = 8.523, Fisher exact test *p* = 0.007). In particular, 18/20 bleeding events (82%) and 4/10 ischemic complications (18%) were documented in the group of patients hospitalized in medical wards. No significant correlation was demonstrated between lung involvement stages and the occurrence of ischemic or hemorrhagic complications in the abdomen (*p* = 0.101). Chest CT also revealed concomitant pulmonary embolism in 9 patients, but the number of cases was inconsistent for an effective statistical analysis.

### Comorbidities

Twenty-five patients (83.3%) had at least one comorbidity. Cardiovascular comorbidities were prominent (23, 76.6%), with arterial hypertension being the most frequent (21, 70%) followed by metabolic (9, 30%) ones, more frequently type-two diabetes mellitus (8, 26.6%) and overweight/obesity (5, 16.6%). Respiratory comorbidities were found in 5 patients (16.6%). Within the 23/30 patients with a known cardiovascular disease, 6/23 (26.1%) suffered an ischemic complication and 17/23 (73.9%) showed a bleeding manifestation. However, no significant correlation was identified between comorbidities and ischemic or hemorrhagic events.

### Laboratory tests

Abnormal laboratory findings in both ischemic and bleeding groups were the following (Table 2): PT INR (median 1.18; 1.08–1.58), aPTT (median 1.23; 1.02–1.73), fibrinogen (median 446 mg/dL; 289–690), neutrophil count (median 12.2 10^9^/L; 8.5–17.4), CRP (median 9.4 mg/dL; 4.5–19.1), IL-6 (median 111 pg/mL; 59–710), procalcitonin (median 0.43 μg/mL; 0.15–3.91) and d-dimer (median 3000 μg/mL; 1367–7740). Platelets and lymphocyte counts were in their normal range. Only d-Dimer levels demonstrated a significant difference between the two groups (*p* < 0.001), being more elevated in the ischemic group. Fibrinogen levels were significantly higher in patients with cardiovascular comorbidities (*p* = 0.047) (Table 3).

**Table 2:** Laboratory parameters in all patients and according to ischemic or bleeding complications

	All patients *N* = 30	Ischemic *N* = 10	Bleeding *N* = 20	*p*
Leucocytes (10^9^/L)	13.4 (10.6–21.0)	13.9 (10.1–18.3)	13.0 (10.5–22.2)	0.713
Neutrophils (10^9^/L)	12.2 (8.5–17.4)	12.9 (7.7–15.6)	11.8 (8.8–20.1)	0.846
Lymphocytes (10^9^/L)	1.4 (0.8–1.6)	1.42 (0.6–3.6)	1.34 (0.8–1.6)	0.880
Platelets (10^9^/L)	242 (172–344)	242 (118–294)	239 (184–356)	0.650
PT-INR	1.18 (1.08–1.58)	1.27 (1.08–1.59)	1.17 (1.08–1.49)	0.456
aPTT	1.23 (1.02–1.73)	1.11 (0.97–2.13)	1.27 (1.12–1.69)	0.604
d-dimer (ng/mL)	3000 (1,367–7740)	8084 (5328–9961)	1771 (1144–3119)	** < 0.001**
Fibrinogen (mg/dL)	446 (289–690)	351 (289–836)	657 (259–657)	0.881
CRP (mg/dL)	9.4 (4.5–19.1)	9.4 (3.2–26.2)	9.2 (4.6–14.1)	0.559
IL-6 (pg/mL)^a^	111 (59–710)	951 (130–7892)	104 (35–193)	0.171
Procalcitonin (μg/mL)	0.43 (0.14–4.31)	2.70 (0.12–20.87)	0.39 (0.15–2.81)	0.397

Data are shown as median (25–75th percentile range). *p* is the statistical significance by Mann–Whitney test between ischemic and bleeding groups

*PT-INR* prothrombin time international normalized ratio, *aPPT* activated partial thromboplastin time, *CRP C*-reactive protein, *IL-6* interleukin-6

^a^IL-6 measurement was available only for 10 patients (4 in ischemic and 6 in bleeding group)

**Table 3:** Clinical and laboratory parameters according to patient’s comorbidities (cardiovascular and no cardiovascular)

	Cardiovascular comorbidities	No cardiovascular comorbidities	*p*
	23	7	
Leucocytes (10^9^/L)	13.2 (10.2–21.6)	13.6 (10.7–17.9)	0.848
Neutrophils (10^9^/L)	11.8 (7.8–19.7)	12.4 (9.8–15.7)	0.962
Lymphocytes (10^9^/L)	1.4 (0.8–1.6)	1.3 (0.6–9.1)	0.848
Platelets (10^9^/L)	251 (190–356)	140 (54–260)	0.158
PT-INR	1.17 (1.08–1.58)	1.26 (1.08–1.58)	0.823
aPTT	1.20 (0.99–1.61)	1.37 (1.19–2.13)	0.181
d-Dimer (ng/mL)	3063 (1184–7882)	3000 (1612–6292)	0.999
Fibrinogen (mg/dL)	586 (352–757)	310 (199–395)	**0.047**
CRP (mg/dL)	9.6 (5.1–22.6)	4.7 (2.7–9.5)	0.077
IL-6 (pg/mL)^a^	106 (45–424)	332 (63-^b^)	0.517
Procalcitonin (μg/mL)	0.43 (0.18–11.9)	0.35 (0.12–1.79)	0.444

Data are shown as median (25–75th percentile range). *p* is the statistical significance by Mann–Whitney test between groups

*PT-INR* prothrombin time international normalized ratio, *aPPT* activated partial thromboplastin time, *CRP* C-reactive protein, *IL-6* interleukin-6

^a^IL-6 measurement was available only for 10 patients (3 in the group without cardiovascular comorbidity and 7 in the group with cardiovascular comorbidity)

^b^75th value not available

### Anticoagulant treatment

The 22 hospitalized patients were under anticoagulant treatment at the time of CT acquisition. In particular, 20 patients started LMWH during hospitalization with therapeutic doses in 12 cases (i.e., enoxaparin, 1 mg/kg twice daily), prophylactic in 4 (enoxaparin 40 mg daily), and intermediate in 1 (enoxaparin, 1 mg/kg daily), respectively. Two patients were already on chronic oral anticoagulant treatment with warfarin. In 3 cases this information was missing. Among the eight ED patients, four were receiving LMWH (1 therapeutic, 2 intermediate, and 1 prophylactic doses), while one was on chronic oral anticoagulant therapy.

As expected, a relation between ischemic/hemorrhagic complications and anticoagulant treatment was noted (*χ*^2^ = 6.667, Fisher exact test *p* = 0.03). Particularly, among the 27 patients on anticoagulant treatment, 7 (26%) had an ischemic complication and 20 (74%) had a hemorrhagic event.

## Discussion

COVID-19 is a systemic disease, with a specific tropism for endothelium cells that leads to microvascular disease, with multisystemic involvement and, therefore, also affecting the gastrointestinal system.

Abdominal ischemic and hemorrhagic manifestations of SARS-CoV-2 have been described in literature since the first pandemic wave [18–20]. Contrast enhanced CT with multiphasic acquisition plays a key role in the diagnosis of these abdominal manifestations and provide to clinicians essential information to assess disease severity in COVID-19 patients. The abdominal ischemic radiological findings are nonspecific, being comparable to other causes of gastrointestinal ischemia [18]. In our cohort of patients with ischemic complications only 1 out of 10 had evidence of acute vessel thrombosis. This may reflect the prevalent small-vessel involvement of COVID-19 disease and is in line with the report from Bhayana et al. [14]. Bleeding manifestations are also frequent, being spontaneous hematomas in soft tissues the most common ones.

COVID-19 coagulopathy could be detected by standard coagulation tests. In accordance with the study of Levi et al. about coagulation abnormalities in patients with COVID-19 [21], d-dimer levels were increased in our population. Furthermore, a significant difference between the ischemic and hemorrhagic cohorts was found, with significantly higher d-dimer levels in ischemic patients. This is likely due to the intense activation of blood coagulation with increased turnover of fibrin production and degradation, which leads to elevated release of fibrin degradation products, including the d-dimer, the final product of cross-linked fibrin lysis. Also, the PT-INR was increased, with a medium value of 1.25 in the whole study population. Approximately 20–50% of COVID-19 hospitalized patients show alterations in coagulation tests, such as high d-dimer, prolonged PT, thrombocytopenia, and low fibrinogen levels [22], suggesting the occurrence of blood clotting activation, similar to disseminated intravascular coagulation (DIC). However, the COVID-19 coagulopathy is different from classical DIC associated with sepsis, in which thrombocytopenia is much more profound and elevation of d-dimer not so intense. This is confirmed by our study, where platelet counts were even in a normal range, supporting the hypothesis that SARS-CoV-2-related DIC is not characterized by significant thrombocytopenia [21].

Is still under debate if bleeding complications are due to the virus induced coagulopathy, as a complication of the anticoagulant therapy or as a combination of the two factors. Right from the beginning of the pandemic, the International Society of Thrombosis and Hemostasis (ISTH) recommended offering prophylactic anticoagulation with low molecular weight heparins (LMWH) to all hospitalized COVID-19 patients as early as possible to prevent thrombotic events and organ damage [16]. However, at that time, emerging data on increasingly higher rates of thrombosis in patients with COVID-19 have led some clinicians to use intermediate or full therapeutic anticoagulant doses, instead of prophylactic doses, in acute and critical patients with COVID-19.

The effect of anticoagulation on bleeding in hospitalized COVID-19 patients is still under investigation. In a study Musoke et al. [23], COVID-19 patients on therapeutic anticoagulation revealed a significantly higher rates of major bleeding compared to those without anticoagulation, while subtherapeutic doses and prophylactic doses did not have any significant differences in major bleeding outcomes compared to patients without anticoagulation. Although associated with risks of bleeding, anticoagulation use has shown to increase survival in patients with severe COVID-19 infection. Differently, a single center retrospective observational study with the aim to identify the value of full therapeutic anticoagulation in patients hospitalized with COVID-19 evaluated 2773 patients with COVID-19: 786 (28%) received therapeutic anticoagulation. In this study, major bleeding was not significantly increased in patients receiving therapeutic anticoagulation (3% therapeutic anticoagulation vs. 1.9% no therapeutic anticoagulation; *p* = 0.2) [24].

In our cohort, all 20 patients with a bleeding manifestation were on anticoagulant therapy. Unfortunately, the dose of anticoagulant therapy was not available for all patients. Without considering 2 patients on OAT, clinical records reported that of the 18 patients in LMWH, 13 followed a therapeutic regimen, 1 an intermediate and 1 a prophylactic one (3 NA). Although we cannot evaluate the incidence of bleeding events in all COVID-19 patients hospitalized in the same period in our Institution, but only in COVID-19 patients who have had an abdominal CT scan for abdominal pain, it is still reasonable to believe that the prevalence of hemorrhagic events over thrombotic ones is noteworthy. Furthermore, a correlation between ischemic or hemorrhagic events with ongoing hospitalization has been pointed out in our study.

Pre-existing comorbidities are related to a general higher risk of severe COVID-19 disease occurrence [25]. In our population, 83.3% of patients had prior comorbidities: in line with data by Callender et al. [26], cardiovascular risk factors were the most common, primarily hypertension, followed by the metabolic factors. Nevertheless, no significant correlation was identified between comorbidities and ischemic or hemorrhagic events occurrence in our study population.

We acknowledge that our retrospective study has several limitations. First, the limited number of patients involved. Another limitation is the different dosage of anticoagulant therapy administered to the patients. We included only patients affected by major events for whom CT was requested; thus, subclinical or non-symptomatic ischemic and hemorrhagic manifestations were ignored. Finally, no specimen or sample testifying the direct relationship among COVID-19 and abdominal complications is available in our cohort. However, all the patients included are from the first Italian peak and this make the correlation strongly likely.

In conclusion, ischemic and haemorrhagic abdominal events are an uncommon but a potentially life-threatening manifestation of COVID-19. When critically ill COVID-19 patients report suggestive clinical symptoms, these complications should be considered and investigated through laboratory and imaging exams. Particularly, CT plays a key role in the early diagnosis of these conditions, helping clinicians in proper management of these patients. Elevated d-dimer is associated with increased risk of abdominal ischemic events. A correlation between therapeutic anticoagulation and these complications was identified, especially with hemorrhagic events. Larger cohort studies are, therefore, necessary to investigate these dangerous COVID-19 manifestations and provide prevention tools.

## Data Availability

Not applicable
